# The Metropolitan Hospital Sunday Fund

**Published:** 1893-12-02

**Authors:** 


### HOSPITAL FESTIVALS AND MEETINGS.

### THE METROPOLITAN HOSPITAL SUNDAY FUND.

A meeting of the Council of this Fund was held in the Mansion House on Friday afternoon, the new Lord Mayor—who was welcomed by Mr. Martin, M.P.—presiding. Of members of the Council there were present the Archdeacon of London, the Chief Rabbi, Canon Ingram, Canon Bristowe, Dr. Kennedy, Mr. Carr-Gomm, Mr. Burdett, and others. The business was for the most part formal. The report of the Council for 1893 was ordered to be published. This report sets forth particulars, which have already been made public, as to the distribution of the sum of £39,290 raised on Hospital Sunday of this year. In it the Council express regret that the total amount of this year's Fund is less than that collected in 1892, apparently caused by the general depression of trade. They are glad, however, to notice that there is no falling off in the number of contributing congregations, which are thirty-one in excess of those of 1892. This report will be submitted at the annual general meeting of constituents of the Fund, which is to be held on December 19th. It was resolved on Friday to recommend that the following names be added to the Council: Lord Knutsford, in room of the late Lord Derby; Sir William Broadbent, in room of the late Sir Andrew Clark; Sir Stuart Knill, Sir Saville Crossley, M.P., the Right Hon. C. T. Ritchie, the Rev. H. M. Paget, and Mr. John Mackrell. Archdeacon Sinclair, the Chief Rabbi, and the Rev. Dr. Kennedy having expressed their approval, it was recommended that Hospital Sunday for 1894 should be held on Sunday, June 10th. Besides the ordinary business of the Fund, especial allusion to the late Sir Andrew Clark was made, he having been most closely connected with the Sunday Fund, and having rendered it signal services. Mr. Carr-Gomm moved that the Council express great regret at the death of Sir Andrew Clark, who had taken a lively interest in the Fund and had pleaded its cause with great earnestness; and that they extend their sympathy and condolence with Lady Clark and the members of the family. All who knew Sir Andrew Clark, said Mr. Carr-Gomm, knew the extraordinary way in which he devoted himself to whatever subject he took in hand—whether at the bedside of a patient, or in the ward of the hospital, or in supporting a resolution at a great public meeting. They who knew him as a friend knew how whole-hearted a friend he was. Although they were so much the poorer by his death, he had attained that euthanasia which he would have desired—of dropping out of life in the midst of work and without the impairing of his faculties. Archdeacon Sinclair seconded in a word. Their loss was inestimable, but Sir Andrew Clark had left a memory from which they might derive inspiration. Mr. Burdett said that this was one of the most momentous losses the fund had ever experienced. As a member of a small committee appointed to endeavour to get pubiic men to help them he (Mr. Burdett) had approached Sir Andrew Clark, and he could only say that Sir Andrew Clark had been one of the most devoted friends of the Hospital Sunday Fund. He hoped that his life would be an example not only to those of them who were laymen but to medical men, on whose efforts and whose eloquence that Fund had the strongest claim. Canon Ingram desired to corroborate what had been said in reference to Sir Andrew Clark, and in a word to speak of what to his own knowledge he had done for the poorer members of the clerical profession.

